# Angiogenesis Inhibition in Prostate Cancer: Current Uses and Future Promises

**DOI:** 10.1155/2010/361836

**Published:** 2010-02-11

**Authors:** Jeanny B. Aragon-Ching, Ravi A. Madan, William L. Dahut

**Affiliations:** ^1^Division of Hematology and Oncology, Department of Medicine, The George Washington University Medical Center, 2150 Pennsylvania Avenue Northwest, Washington, DC 20037, USA; ^2^Laboratory of Tumor Immunology and Biology, National Cancer Institute, National Institutes of Health, Bethesda, MD 20892, USA; ^3^Medical Oncology Branch, National Cancer Institute, National Institutes of Health, Bethesda, MD 20892, USA

## Abstract

Angiogenesis has been well recognized as a fundamental part of a multistep process in the evolution of cancer progression, invasion, and metastasis. Strategies for inhibiting angiogenesis have been one of the most robust fields of cancer investigation, focusing on the vascular endothelial growth factor (VEGF) family and its receptors. There are numerous regulatory drug approvals to date for the use of these agents in treating a variety of solid tumors. While therapeutic efficacy has been established, challenges remain with regards to overcoming resistance and assessing response to antiangiogenic therapies. Prostate cancer is the most common noncutaneous malignancy among American men and angiogenesis plays a role in disease progression. The use of antiangiogenesis agents in prostate cancer has been promising and is hereby explored.

## 1. Introduction

Angiogenesis is a complex, dynamic process that involves multiple pathways that converge to affect carcinogenesis, proliferation, and tumor growth. Since the inception of the concept of angiogenesis by Dr. Judah Folkman decades ago, a substantial body of research has emerged and currently forms the groundwork for establishing antiangiogenesis as an important part of the armamentarium in cancer therapy. Furthermore, investigation of signaling pathways, molecules, drugs, as well as mechanisms of resistance may lead to a better understanding of angiogenesis and the development of strategies incorporating antiangiogenic drugs with chemotherapy in various tumor types, such as prostate cancer.

Prostate cancer is the leading noncutaneous malignancy among American men in North America. In 2009 alone, it is estimated that 192,280 new cases will be diagnosed and more than 27,000 deaths will occur from this disease [1]. Hormonal therapy remains the cornerstone of treatment for men who have androgen-responsive metastatic disease. While most men will respond to sequential hormonal manipulations, castration resistant prostate cancer (CRPC) eventually ensues. The demonstration of survival benefit using docetaxel-based therapy [2, 3] led to the approval by the Food and Drug Administration (FDA) in 2004 of docetaxel and prednisone for the treatment of metastatic CRPC. Since then, no other drug has been approved for metastatic CRPC, thus creating an area of increased unmet medical need.

Extensive studies on angiogenesis in prostate cancer to date have revealed that angiogenesis plays a role in the progression of prostate cancer. Microvessel density, a measurement of prostate cancer angiogenesis, has been shown to be a predictor of metastasis and survival [4]. Thus, targeting angiogenesis has been the subject of several clinical investigations.

## 2. Pathways Involved in the Angiogenic Process

Since the introduction of the concept that tumors would not grow beyond a pinhead size in the absence of blood vessel growth [5], our understanding of the role that angiogenesis plays in cancer has robustly expanded. Angiogenesis is a complex process that involves an interplay between various regulatory proteins, proangiogenic stimuli, endothelial cell activation, as well as proliferation and migration, governed by molecular and cellular mechanisms, resulting in reorganization into new blood vessels [6]. The theory of the “angiogenic switch” describes the dynamic transition to a malignant tumor phenotype that promotes neovascularization, the absence of which is a rate-limiting step in carcinogenesis [7–10]. Several factors may trigger proangiogenic factor expression. Hypoxia, for instance, regulates the production of several angiogenic cytokines such as fibroblast growth factor 2 (FGF-2), vascular endothelial growth factor (VEGF), transforming growth factor-beta (TGF-*β*), tumor necrosis factor-alpha (TNF-*α*), and interleukin-8 (IL-8), among others. The overall tumor microenvironment is also instrumental in the recruitment of proangiogenic factors [11], although the resulting tumor neovasculatures are characterized by inefficient, permeable, and leaky vessels [12].

**Vascular Endothelial Growth Factor Pathway (VEGF)**

Perhaps the most widely studied pathway in the angiogenic signaling process involves triggering VEGF and its receptors. The VEGF family has more than 7 members, including VEGF-A, VEGF-B, VEGF-C, VEGF-D, VEGF-E, PlGF, and *Trimeresurus flavoviridis* (*T.f*.) svVEGFs [13]. VEGF-A is the prototype VEGF ligand, playing a key role in tumor angiogenesis. VEGF-A binds and activates two receptors, namely, VEGFR1 (Flt-1 or fms-like tyrosine kinase receptor 1) and VEGFR2 (KDR/Flk-1), and it has varying roles in the promotion of endothelial cell differentiation, cell growth, tubular formation, and migration [14]. Neutralization of the VEGF-A ligand has, therefore, been the subject of investigation in the last decade, leading to the first FDA drug approval of its class, Bevacizumab (Avastin; Genentech, Inc., South San Francisco, CA). Other strategies for targeting the VEGF pathway involve the inhibition of key enzymes such as tyrosine kinase inhibitors or use of decoy receptor fusion proteins.

## 3. Prostate Cancer: Experience with Use of Antiangiogenic Agents

Early studies yielded promising results of VEGF inhibition in various murine tumor models [15, 16]. In prostate cancer, elevated circulating VEGF and other soluble growth factors have been demonstrated to be predictive of biochemical progression in men undergoing radical prostatectomy [17]. Measurements of angiogenesis using microvessel density have also been shown to be a prognosticator for survival and metastasis [4, 18, 19]. The utility of microvessel density has also been shown to improve prediction of cancer staging from prostate biopsies, beyond the well-known contributory factors afforded by existing risk features such as Gleason scores and prostate-specific antigen (PSA) levels [20]. In a prospective study among 572 men diagnosed with prostate cancer in the Health Professionals Follow-Up Study, those with the most irregular and primitive microvessel density as measured by staining CD34 were more likely to have lethal and aggressive prostate cancer [21]. This section will discuss varying agents used in the treatment of prostate cancer including thalidomide, bevacizumab, and the tyrosine kinase inhibitors.

### 3.1. Thalidomide

Thalidomide (alpha-N-phthalimidoglutarimide; Thalomid, Celgene, Summit, NJ) has emerged as a potent treatment for several disease entities and has been currently approved by the FDA in the United States, including for the treatment of Multiple Myeloma [22]. Although its antiangiogenic properties are not clearly understood, several in vitro assays have suggested that the antiangiogenic properties could be secondary to the inhibition of secretion of two angiogenic cytokines, namely, VEGF and FGF from both tumor and stromal cells [23, 24]. Evidence to date suggests that this occurs independently of thalidomide's immunomodulatory properties and that the downregulation of integrins perhaps results in the inhibition of endothelial cell migration and adhesion [25]. Although thalidomide has predominantly been studied in hematologic malignancies, activity in solid tumors has also been demonstrated. Thalidomide in prostate cancer has been used alone or in combination with chemotherapy in CRPC. An earlier study using thalidomide in an open-label phase II randomized trial compared low-dose (200 mg/day) and high-dose (up to 1200 mg/day) thalidomide in 63 patients [26]. Results showed a modest response with 27% of the patients having a reduction in the serum PSA levels of ≥40%. Four patients in the low-dose arm showed sustainable response of >150 days with a >50% decrease in PSA. The demonstration of the potential activity of thalidomide and preclinical evidence demonstrating that chemotherapy could enhance the activity of antiangiogenic agents [27] led to the combination of thalidomide with docetaxel [28]. This randomized phase II study of docetaxel, with or without 200 mg of thalidomide, enrolled 75 patients with chemotherapy-naive metastatic CRPC. Docetaxel, at a dose of 30 mg/m^2^ given intravenously, was administered weekly for 3 out of 4 weeks, with a 1-week rest period during the 4th week. The trial was launched prior to the TAX 327 trial which demonstrated the superiority of docetaxel every 3 weeks; thus, the weekly dose was administered. PSA declines of at least 50% were greater in the combined docetaxel and thalidomide arm compared to the docetaxel alone arm, with better progression-free survival (PFS) which was not statistically significant (median PFS of 3.7 months in docetaxel group and 5.9 months in the combined group (*P* = .32)) [29]. The side effects were manageable although thrombotic events were seen in the combination arm which was later alleviated by institution of thromboprophylaxis. Further overall survival analysis of this trial showed an improvement in the 18-month survival in the combination arm versus docetaxel alone arm (69.3% versus 47.2%, *P* < .05) [30].

To this end, a search for a more efficacious and less toxic thalidomide analog such as lenalidomide was studied in prostate cancer. A phase I study to determine the maximum tolerated dose (MTD) and to characterize the side-effect profile and pharmacokinetics (PKs) of lenalidomide in patients with advanced refractory solid tumors was launched [31]. Forty-five patients were enrolled, of whom 36 patients had prostate cancer. Dose levels used were 5 mg, 10 mg, 15 mg, 20 mg, 25 mg, 30 mg, 35 mg, and 40 mg. The dosing schedule was modified from continuous daily to 21 out of 28 days of dosing due to the observed side effects. Interestingly, stable disease was seen in 12 out of 44 evaluable patients, 9 of whom had prostate cancer. A phase I dose-escalation trial using docetaxel at 60 mg/m^2^ and 75 mg/m^2^every 21 days, combined with lenalidomide at varying doses from 10 mg to 30 mg on days 1–21, showed promising responses with manageable toxicity [32]. There were 31 patients evaluable for PSA response. PSA declines of >50% were seen in 47% (8 out of 17) previously untreated patients and 50% (7 out of 14) previously treated patients. The promising activity seen in prostate cancer, as well as the demonstrated activity using thalidomide, has led to an ongoing phase II trial using the combination of lenalidomide, docetaxel, prednisone, and bevacizumab, in chemotherapy-naive patients with metastatic CRPC at the National Cancer Institute [33].

### 3.2. Bevacizumab

Bevacizumab is the most widely studied antiangiogenic inhibitor and the first of its class to be approved by the United States FDA. It has gained approval predominantly in combination with chemotherapy for metastatic colorectal cancer, lung, breast, renal cell cancer, and Glioblastoma multiforme [34]. Bevacizumab is a humanized IgG1 monoclonal antibody developed from a murine monoclonal antibody targeting the human VEGF ligand, specifically the major isoforms of VEGF-A. The initial studies using a murine monoclonal antibody targeting VEGF on several human cancer cell lines in vivo showed promising results [15, 35]. However, initial single agent studies in prostate cancer were disappointing[36]. Fifteen patients with metastatic CRPC were treated at a dose of 10 mg/kg of bevacizumab every 2 weeks for 6 total infusions. No objective or partial responses were observed by day 70 in the 8 patients who had measurable disease. Only 4 patients had PSA declines, none more than 50%. Although single-agent bevacizumab lacked significant activity in prostate cancer, as in most other solid tumors, the encouraging results from other clinical trials using combined bevacizumab and chemotherapy led to a Cancer and Leukemia Group B (CALGB) trial 90006 that combined bevacizumab with docetaxel and estramustine [37]. The CALGB 90006 trial enrolled 79 patients, and a 77% PSA decline rate of 50% was observed (in the 58 of 75 patients with sufficient PSA data) [37, 38]. The estimated time to progression was 9.7 months and the overall survival was 21 months.

Similar promising results were shown in a phase II, open-label trial at the National Cancer Institute (NCI) utilizing a combination of bevacizumab at 15 mg/kg every 3 weeks, in combination with docetaxel 75 mg/m^2^ every 3 weeks, thalidomide 200 mg daily, and prednisone 10 mg daily, with thromboprophylaxis using a low-molecular weight heparin. This trial enrolled 60 patients with metastatic CRPC, with a median age of 66 years (range 44–79), and predominantly high-risk features, including a median Gleason score of 8, on-study PSA of 99 ng/mL (range: 6.0–4,399), and prestudy PSA doubling time of 1.6 months (0.3–18.2, 81% <3 months). As of the last followup [39], 51 patients (88%) had PSA declines of >50%, with median >50% PSA decline duration of 11 cycles (0–45). Furthermore, the overall response rate was 63% of the 32 patients with measurable disease, with a complete response (CR) seen in 2 patients, partial response (PR) in 18 patients, and stable disease (SD) in 11 patients. This combination was tolerable with expected adverse-effects that included febrile neutropenia in 5 patients, syncope in 5 patients, gastrointestinal perforation or fistula in 3 patients, and thrombosis or bleeding in 5 patients. The estimated median PFS was 18.2 months.

Another CALGB study that evaluated bevacizumab in prostate cancer was CALGB 90401 which had the primary objective of comparing overall survival between men with chemotherapy-naive metastatic CRPC treated with standard of care docetaxel 75 mg/m^2^ every 21 days and prednisone 5 mg twice daily versus docetaxel 75 mg/m^2^, prednisone 5 mg twice daily, and bevacizumab 15 mg/kg every 21 days. The study completed accrual in December 2007 and results are currently awaited. The study was powered to detect a 25% improvement in overall survival in the bevacizumab arm [40].

### 3.3. VEGF Trap

Another strategy for targeting VEGF is through blocking the VEGF receptors. One of the most potent VEGF-R blockers is a novel decoy receptor fusion protein comprised of the extracellular domain 2 of VEGFR-1 and domain 3 of VEGFR-2 fused to the constant region (Fc fragment) of human IgG1 [41]. Earlier studies using truncated soluble VEGF-R1 inhibitors exhibited effective inhibition of VEGF but had poor pharmacokinetic profile and had to be administered more frequently and at high concentrations [42, 43]. VEGF Trap (Aflibercept; Sanofi Aventis, Paris, France and Regeneron, Tarrytown, New York) is a human fusion protein that binds and neutralizes the major VEGF isoforms including VEGF-A, VEGF-B, as well as platelet-derived growth factor (PlGF) [44]. A phase I dose escalation study using aflibercept in combination with docetaxel 75 mg/m^2^ every 3 weeks has been reported [45], with recommended dosing of aflibercept at 6 mg/kg. A phase III trial has been launched in metastatic CRPC patients with a primary objective of improvement in overall survival for metastatic CRPC and a planned accrual of 1,200 patients who will be randomized to either VEGF Trap in combination with standard docetaxel and prednisone or standard docetaxel and prednisone alone [46].

### 3.4. Tyrosine Kinase Inhibitors

Tyrosine kinases are key enzymes that modulate various cellular processes that affect signaling for tumor growth, proliferation, and survival [47]. Several tyrosine kinase inhibitors have been used in the treatment of prostate cancer. Sorafenib (Nexavar; Bayer HealthCare Pharmaceuticals Inc., Wayne, NJ) and sunitinib (Sutent; Novartis, East Hanover, NJ) lead the agents that have been used. Sorafenib not only inhibits VEGF but functions as a multi-tyrosine kinase inhibitor that has been shown in preclinical models to inhibit wild-type and mutant b-Raf and c-Raf kinase isoforms in vitro. In addition, this agent also inhibits various pathways, including p38, c-kit, VEGFR-2, and PDGFR-*β* in varying concentrations, affecting tumor growth as well as possibly promoting apoptosis by events downstream of c-Raf [48, 49]. Clinical studies using sorafenib have been done in various phase II studies but have shown only modest activity and no robust PSA declines. In a phase II study using sorafenib in 22 patients, no PSA declines of 50% were noted [50]. However, there was discordance between PSA rise and improvement in bone lesions by bone scintigraphy scan in two patients. This led to further accrual of the trial to the accrual goal of 46 patients [51]. Other phase II studies using sorafenib showed similar minimal PSA activity in spite of radiographic improvements [52–56].

Sunitinib malate is another small molecule inhibitor targeting VEGFR-1 and VEGFR-2, along with PDGF-R, c-kit, and *RET* kinases [57]. This agent has been shown to exhibit some activity in prostate cancer. However, similar to sorafenib, sunitinib exhibited few PSA declines and several patients had clinical and radiographic improvements despite PSA rises [58]. The use of these agents, therefore, raises the question of whether adequate assessments are being used in analyzing the treatment effects using these multikinase inhibitors or perhaps they are best combined with other agents, such as chemotherapy.

Cediranib (Recentin; AstraZeneca, London, UK) is another agent that inhibits tyrosine kinases of VEGF receptors. This is an oral, potent, indole-ether quinazoline ATP-competitive compound that inhibits proliferation via inhibition of all 3 VEGF receptors [59]. A phase I study using cediranib in prostate cancer showed dose-limiting toxicity occurring at 30 mg dose, with a dose of 20 mg/day being the MTD [60]. A phase II study in docetaxel-resistant metastatic CRPC showed encouraging results, with 13 of 23 evaluable patients having decreases in soft tissue lesions, 4 of whom met criteria for partial response [61]. Shrinkage of metastatic visceral disease to the lymph nodes, lung, liver, and bone was observed although the PSA levels have not corresponded with imaging responses. Similar to sorafenib, posttreatment PSA declines were noted in patients following drug discontinuation, in the absence of administration of a new drug treatment.

## 4. Mechanisms of Resistance

It was initially believed that angiogenesis inhibition carries little potential risk for resistance [62]. However, recent studies suggest that this may not be the case. For instance, experiments using mouse endothelial cells isolated from human tumor xenografts when compared to normal endothelial cell counterparts showed acquired cytogenetic abnormalities [63]. Furthermore, regrowth of tumors during treatment with antibodies to VEGFR1 and R2 after an initial period of growth suppression was seen in a pancreatic islet cell tumor murine model, suggesting development of phenotypic resistance to VEGFR2 blockade [64]. This resistance to VEGF blockade involves various possible mechanisms, including an adaptive evasion or intrinsic nonresponsiveness of tumors [10, 65]. Adaptive evasion suggests upregulation of alternative signaling pathways to circumvent the blocked angiogenic pathway, recruitment of bone-marrow derived proangiogenic cells, or increased surrounding pericyte coverage. Intrinsic nonresponsiveness, on the other hand, suggests innate indifference of the tumor to antiangiogenic therapy, which supports the clinical observation that not all patients respond to antiangiogenic therapy. These observations certainly have clinical implications since strategies to obviate these acquired or inherent resistances must be sought. Strategies that include combination or sequential approach of antiangiogenic therapies may be one approach to address this. In addition, genetic variability in the VEGF promoter can be used as a potential predictive biomarker for bevacizumab activity.

## 5. Future Directions

Angiogenesis inhibitors have the potential to enhance therapeutic options for patients with prostate cancer. Various combinations, not limited to chemotherapy, have been used with promising results. For instance, interesting results have been seen using a combination of bevacizumab with the autologous dendritic cell-based vaccine sipuleucel-T (Provenge; Dendreon, Seattle, WA) in patients with biochemical recurrence [66]. This vaccine has demonstrated improved overall survival relative to placebo in a phase III trial in metastatic CRPC [67] and may warrant further investigation with bevacizumab. With the success seen in various other tumor types using antiangiogenesis, similar results are likely seen in prostate cancer. However, several challenges remain, including the use of appropriate assessment tools for measuring response in metastatic prostate cancer and the use of candidate surrogate biomarkers for correlating response. Perhaps the use of pharmacogenetics may help in identifying patients who may benefit or develop undue toxicity from the use of antiangiogenic agents. For instance, in a trial using bevacizumab in breast cancer patients, varying single nucleotide polymorphisms (SNPs) in the regulatory regions of the VEGF gene may predict for improved median overall survival or protection from hypertension, which is one of the most commonly encountered toxicity with the use of bevacizumab [68]. The benefit of angiogenesis inhibitors has become a reality in several tumor types, with significant potential in prostate cancer.
